# Integrative data modeling from lung and lymphatic cancer predicts functional roles for miR-34a and miR-16 in cell fate regulation

**DOI:** 10.1038/s41598-020-59339-y

**Published:** 2020-02-13

**Authors:** Shantanu Gupta, Daner A. Silveira, Florencia M. Barbé-Tuana, José Carlos M. Mombach

**Affiliations:** 1 Departamento de Física, Universidade Federal de Santa Maria, Santa Maria, RS Brazil; 2 Postgraduate Program in Cellular and Molecular Biology, Pontifícia Universidade Católica do Rio Grande do Sul, Porto Alegre, Brazil

**Keywords:** Computational biology and bioinformatics, Systems biology

## Abstract

MiR-34a and miR-16 coordinately control cell cycle checkpoint in non-small cell lung cancer (NSCLC) cells. In cutaneous T-cell lymphoma (CTCL) cells miR-16 regulates a switch between apoptosis and senescence, however the role of miR-34a in this process is unclear. Both miRNAs share many common targets and experimental evidences suggest that they synergistically control the cell-fate regulation of NSCLC. In this work we investigate whether the coordinate action between miR-34a and miR-16 can explain experimental results in multiple cell lines of NSCLC and CTCL. For that we propose a Boolean model of the G1/S checkpoint regulation contemplating the regulatory influences of both miRNAs. Model validation was performed by comparisons with experimental information from the following cell lines: A549, H460, H1299, MyLa and MJ presenting excellent agreement. The model integrates in a single logical framework the mechanisms responsible for cell fate decision in NSCLC and CTCL cells. From the model analysis we suggest that miR-34a is the main controller of miR-16 activity in these cells. The model also allows to investigate perturbations of single or more molecules with the purpose to intervene in cell fate mechanisms of NSCLC and CTCL cells.

## Introduction

Non-small cell lung cancer (NSCLC) is responsible for most of the cancer-related deaths worldwide, making it a major medical issue and an interesting target for drug development. The dysregulation of potent signaling pathways through alterations in key oncogenes and tumor suppressors is one of the hallmarks of NSCLC, leading to uncontrolled cell proliferation and survival. On the other hand, recent studies identified new pathways that can control cell proliferation in NSCLC through downstream DNA damage mechanisms. In this context, the Bandi and Vassella^1^ study demonstrated that a synergistic action between microRNAs (miRNAs) in response to DNA damage can be effective to block proliferation in NSCLC cells. miRNAs are small non-coding RNAs that are frequently involved in many biological processes such as cancer progression. This study has focus on this synergistic function between microRNA-34a (miR-34a) and microRNA-16 (miR-16) in cell fate decision of NSCLC cells. These molecules are well known master regulators of tumor suppression and play an important role in DNA-damage response through induction of cell cycle arrest and apoptosis.

MiR-34a and miR-16 are down-regulated in several types of cancers including NSCLC^1^ and cutaneous T-cell lymphoma (CTCL)^2,3^. MiR-34a and miR-16 are regulators of a variety of different molecules related to cell cycle progression such as the cyclin-dependent kinase 4 and 6 (CDK4 and CDK6), allowing the coordinated regulation of cell fate decision. Although both miRNAs contain completely different seed sequences, they are functionally related since they are both able to induce the G1/S cell cycle checkpoint^1,4,5^. These molecules can enhance the cyclin-dependent kinase inhibitor 1 A (CDKN1A,p21) expression, in NSCLC cells miR-34a is able to directly suppress the Myc proto-oncogene protein (Myc)^5^ and the Histone deacetylase 1 (HDAC1)^6^ as both negatively regulate p21. Similarly, miR-16 directly inhibits the proto-oncogene, polycomb ring finger (BMI1) increasing p21 activity^2^. However, the role of the coordinated regulation of cell fate decision involving miR-34a and miR-16 influence is not completely characterized yet in NSCLC and CTCL cells.

In the present study we model the regulatory role and synergistic action^1^ of miR-16 and miR-34a in the G1/S cell cycle checkpoint using Boolean methods^7–10^.

## Methods

### The molecular mechanisms of the G1/S checkpoint in NSCLC and CTCL cells

**Figure 1:**
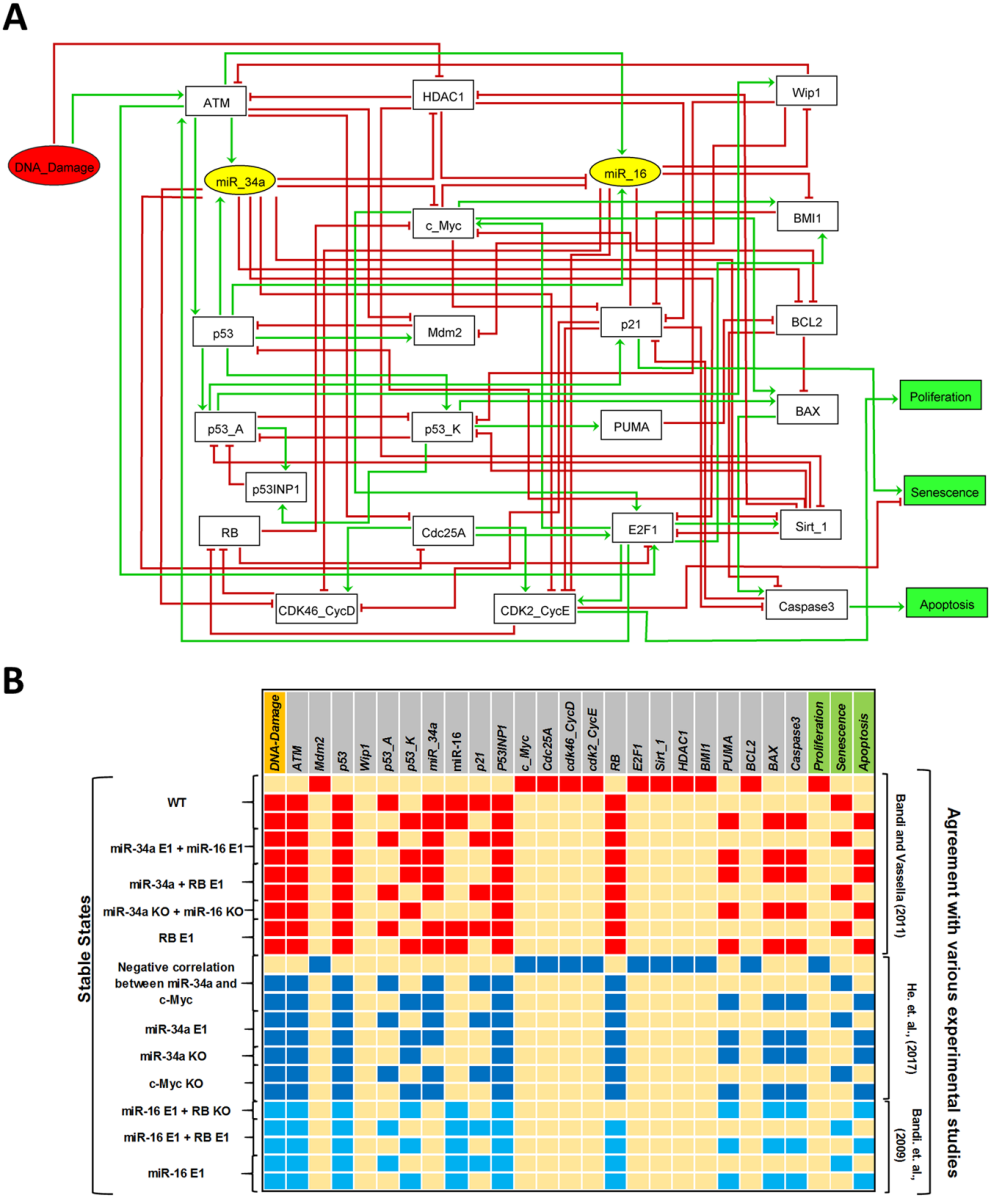
Synergistic model for NSCLC and corresponding simulation results. (**A**) Regulatory network for G1/S checkpoint in response to DNA damage. Rectangular nodes represent proteins and the two yellow elliptic nodes represent miR-34a and miR-16. The red input denotes DNA damage, whereas rectangular green nodes represent model outputs (Proliferation, Senescence and Apoptosis). Green and red arcs denote activatory and inhibitory interactions, respectively. (**B**) Wildtype case states and the result of Gain-of-Function (GoF) and Loss-of-Function (LoF) perturbations corresponding to referential experiments. Ectopic expression (E1) represents a GoF and Knock-out (KO) represents LoF of the corresponding element. Yellow cells denote a null value, whereas red, dark blue and light blue cells denote value 1, respectively.

The proposed logical rules that control the nodes in our model are based on the biochemical literature cited in Supplementary Table S1. We define our Boolean model of the G1/S cell cycle checkpoint contemplating the interactions of miR-34a and miR-16. (see Fig. 1A). Cell fate determinations happen at checkpoints^11,12^. In NSCLC cells, miR-34a and miR-16 regulate the induction of the G1/S checkpoint, in fact, NSCLC cells knockout for miR-34a and miR-16 cannot arrest cells at G1/S^1^. The model has a single input, DNA damage (Fig. 1A). DNA double-strand breaks (DSBs) produced as an effect of ionizing radiation^13^ cause the autophosphorylation of ATM serine/threonine kinase (ATM) at serine 1981 initiating its kinase activity^14^. Downstream phosphorylations at the ATM pathway lead to the activation of p53 in response to DNA damage. The ATM autophosphorylation property is crucial for repair of damaged DNA and/or for apoptosis^14^. Radiation directly inhibits HDAC1 which is upregulated in NSCLC^15,16^ and CTCL cells^17^. HDAC1 regulates DNA damage response and directly inhibits ATM expression^18^. ATM can directly induce miR-34a and miR-16 expression^19–21^ and HDAC1 is a direct target of miR-34a^22^, thereby, miR-34a enhances ATM expression by directly inhibiting HDAC1. After DNA damage, ATM phosphorylates p53 which transactivates miR-34a and miR-16^23,24^ expression. For more information about miR-34a targets, see the review by Lacombe^25^ and about miR-16 targets, see ref. ^26^. Activated p53 triggers transcription of E3 ubiquitin protein ligase (Mdm2)^27^. In the model, based on the different phosphorylation states that p53 possess, it is further represented by two other variables: p53-A and p53-K. P53-A represents p53 phosphorylated at serine 15 and serine 20, whereas p53-K describes an additional phosphorylation at serine 46 (for more details about p53-A/p53-K function see^7,28^). P21, protein phosphatase, Mg2 + /Mn2 + dependent 1D (Wip1) and tumor protein p53 inducible nuclear protein 1 (TP53-INP1) are activated by p53-A. Wip1 dephosphorylates ATM serine/threonine kinase (ATM). This action makes ATM induce p53 to the p53-A form that activates transcription of Wip1, which in turn inactivates ATM^29^. Whereas, Bcl-2-binding component 3 (BBC3, PUMA) and BCL2 associated X, apoptosis regulator (BAX) are activated by p53-K^30^.

### The Boolean formalism

The approach is based on the definition of a regulatory graph, where each node represents a molecule and each directed arc (or edge) represents an activation or an inhibition between two nodes^31^. The nodes are Boolean variables taking only 0 (OFF) and 1 (ON) values. Based on the interpretation of biochemical information, a logical rule is assigned to each node in the network, which determines its activation level in terms of the state of its regulators. The logical rules are built using the logical operators AND, OR, and NOT (see Supplementary Table S1 along with the official names of molecules and the biochemical literature). The dynamical behavior of a Boolean model can be represented by a state transition graph (STG). In this graph, each node represents the state of all variables of the network and the arcs denote transitions between states^32^.

Using in silico Gain or Loss-of-Function (GoF/LoF) perturbations, we force node values to remain ON or OFF, respectively, to test the influence of specific nodes and circuits on the dynamics of the network and resulting phenotypes. Arc deletions can also be used for the same purpose. Circuits, which are closed paths on the network where information follows in the same direction, can be positive or negative. Positive circuits have an even number of inhibitions along their path, while negative have an odd number. Positive circuits control stable (or fixed) states of the network associated to defined phenotypes, while negative ones induce cyclic behavior associated with transient phenotypes^33^. The circuit influence on the network dynamics may or not happen depending on its functionality context^32^. Here we consider only the asynchronous update of the network^34^, which has the potential to describe non-deterministic behavior observed in molecular networks.

We built our Boolean network using GINsim 3.0.0b, which is a Java software suite freely available for download from (http://www.ginsim.org/downloads)^35^. The network was built based on the scientific literature and from databases such as BioGRID 3.5 (https://thebiogrid.org/)^36^, TargetScanHuman 7.1 (http://www.targetscan.org/vert_71/)^37^ and miRTargetLink (https://ccb-web.cs.uni-saarland.de/mirtargetlink/index.php)^38^. GINsim tools help to identify the circuits and their functionality, attractors and their reachability probabilities^32^. The GINsim model file can be found in the Supplementary file S1.

## Results

### Phenotypic characterization of the wild-type network phenotypes

The final network in Fig. 1 and the logical rules in Supplementary Table S1 integrate experimental information concerning miR-34a and miR-16. The regulatory roles were mainly based on the work by Bandi and Vassella propose a synergistic action of both miRNAs in cancer^1^.

The wild type case (WT) presents 3 attractors corresponding to different phenotypes (Fig. 1B). The first state (when DNA damage = 0) is a proliferative stable state meaning that checkpoint arrest is not induced as only cell cycle promoters are activated (E2F1, cdk46_CycD, cdk2_CycE, c-Myc, Cdc25A, Sirt_1, BMI1 and BCL2). The second and third states (when DNA damage = 1) correspond to cycle arrest with a stochastic fate decision between Senescent and Apoptotic phenotypes controlled by the positive circuit involving p53-A and p53-K. This type of dynamics is also known as bistable state.

### *In silico* modeling versus *in vitro* knowledge

In Table 1 we list the experiments used as major references to our study. The procedure used to construct our model was to set the model to correspond exactly to the information provided by each study, for example, two studies focused in one miRNA only, ignoring the other^4,5^ (see Fig. 1B).

**Table 1:** Experimental studies used to develop our model.

Experimental studies	miRNAs were used in these studies	Model test	Cancer/Cell lines
Bandi and Vassella^1^	miR-34a and miR-16	red color in Fig. 1B	NSCLC (A549)
Bandi *et al*.^4^	miR-16	light blue color in Fig. 1B	NSCLC (A549)
He *et al*.^5^	miR-34a	dark blue color in Fig. 1B	NSCLC (A549)
Kitadate *et al*.^2^	miR-16	Fig. 3B	CTCL (MyLa, MJ)
?	miR-34a		CTCL (MyLa, MJ)
?	miR-34a and miR-16		CTCL (MyLa, MJ)

Studies for which no experimental data were found are indicated by question marks.

In addition to the evidences related to the synergistic mechanism of miR-34a and miR-16 in NSCLC cells^1^, these molecules were also analyzed in terms of their individual roles in the NSCLC cell model. To do this, the analysis of the individual regulatory role of miR-34a was performed considering miR-16 LoF and for miR-34a we did the opposite. Another used experimental information is that miR-34a expression presents a strong positive correlation with senescence in NSCLC cells via inhibition c-Myc and/or HDAC1 expression^5,22^.

Having the model corroborated by the referential studies, then we decided to expand its validation to other cell lines (see Table 2) and to make predictions about perturbations which were not found in the literature (question marks in Table 2). For example, our model predicts that miR-16 induces Senescence in CTCL cells in RB-dependent manner and that the overexpression or knockdown of RB directly affects miR-16 expression. Another instance of the predictions is that, under DNA damage, the knockdown of Sirt1 and HDAC1 induces senescence or apoptosis in CTCL cells. For more details about model validation see Supplementary file S2.

**Table 2:** Agreement between proposed logical model and experimental data from the literature.

Stimulus/Perturbations	Response/Phenotype	Cell lines	Reference
miR-34a in NSCLC	Downregulated	A549, H460	^5^
In response to DNA damage, miR-34a in NSCLC	upregulated	A549, H460	^5^
miR-34a and c-Myc in NSCLC	Negative correlation	A549, H460	^5^
miR-34a KO in NSCLC	Apoptosis	A549, H460	^5^
miR-34a E1 in NSCLC	Inhibits Proliferation/induces Senescence and Apoptosis	A549, H460	^5^
c-Myc KO in NSCLC	Senescence and Apoptosis	A549, H460	^5^
E2F1 KO in NSCLC	Senescence and/or Apoptosis	A549, H460	?
Sirt-1 KO and E2F1 KO in NSCLC	Senescence and/or Apoptosis	A549, H460	?
miR-16 E1 and RB KO in NSCLC	Apoptosis	A549, H460	^4^
miR-16 E1 and RB E1 in NSCLC	Senescence and/or Apoptosis	A549, H460	^4^
miR-16 E1 in NSCLC	Senescence and/or Apoptosis	A549, H460	^4^
miR-34a and miR-16 in NSCLC	Downregulated	A549, H460	^1^
In response to DNA damage, miR-34a and miR-16 in NSCLC	Upregulated	A549, H460	^1^
miR-34a E1 and RB E1	Senescence	A549, H460	^1^
miR-34a E1 and miR-16 E1	Senescence and/or Apoptosis	A549, H460	^1^
miR-34a KO and miR-16 KO	Apoptosis	A549, H460	^1^
miR-16 in CTCL	Downregulated	MyLa, MJ	^2^
In response to DNA damage, miR-16 in CTCL	Upregulated	MyLa, MJ	^2^
miR-16 and BMI1 in CTCL	Negative correlation	MyLa, MJ	^2^
miR-16 KO in CTCL	Apoptosis	MyLa, MJ	^2^
miR-16 E1 in CTCL	Senescence and/or Apoptosis	MyLa, MJ	^2^
BMI1 KO in CTCL	Senescence and/or Apoptosis	MyLa, MJ	^2^
RB KO in CTCL cell	Directly affects miR-16 expression	MyLa, MJ	?
RB E1 in CTCL cell	Increased miR-16 expression	MyLa, MJ	?
Sirt-1 E1 in CTCL cell	Apoptosis	MyLa, MJ	?

E1 represents GoF and KO represents LoF of the corresponding gene. Question marks indicate predictions of the model since we did not find experimental data to support it.

### Towards the control of miR-16 by miR-34a in NSCLC or CTCL cells

Previously, Bandi and Vassella^1^ showed that miR-34a and miR-16 co-regulated in synergistic and RB-dependent manner. They further demonstrated that both miRNAs (miR-34a and miR-16) share the same targets in NSCLC cells. However, it does not mean that there is some functional link between these two miRNAs. In such manner, the study of Bandi and Vassella^1^ could not reveal the role of miR-34a and miR-16 in a synergistic manner in NSCLC.

**Figure 2:**
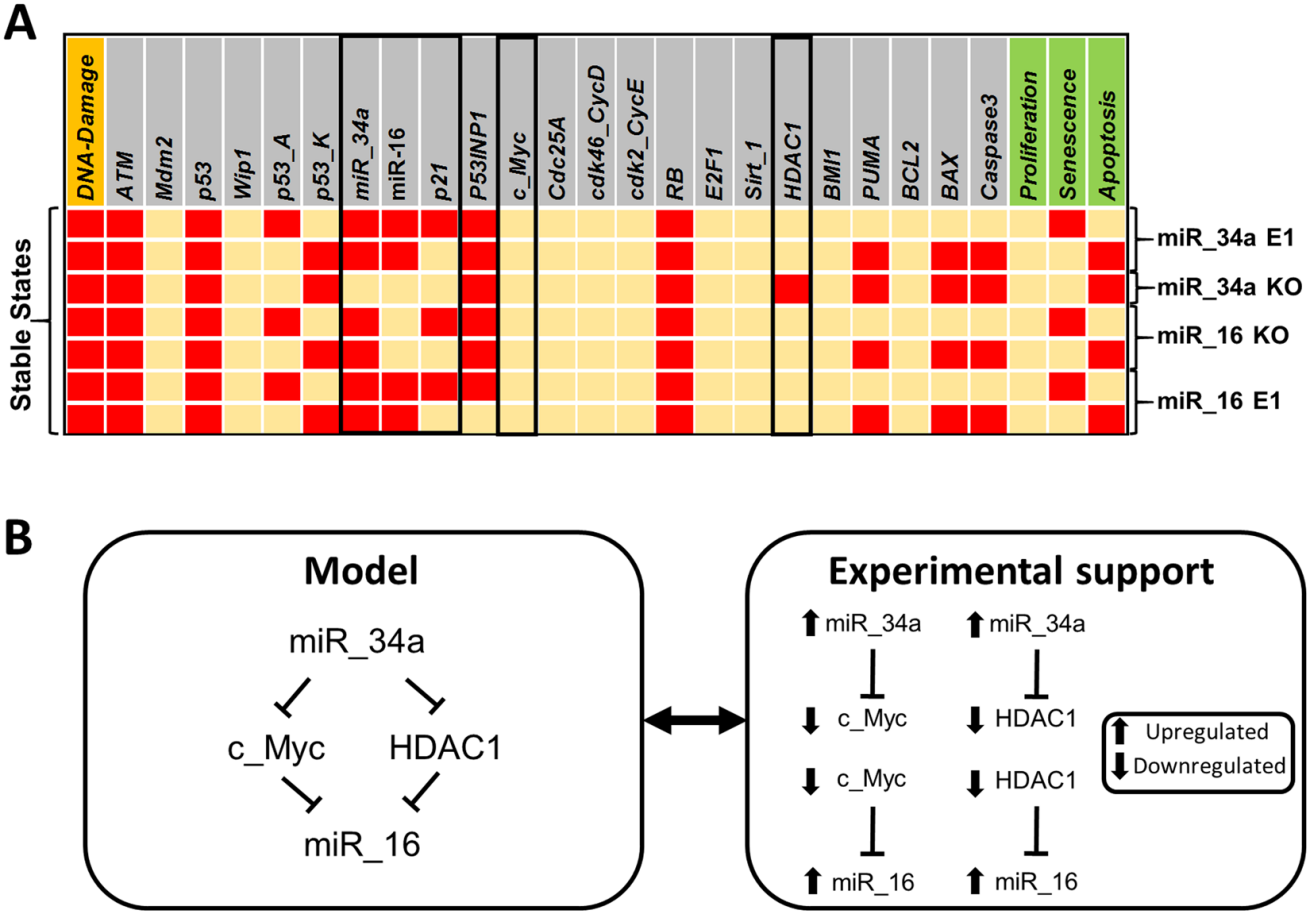
MiR-34a regulation of miR-16 expression. (**A**) The stable states identified for distinct scenarios of the miRNAs activity: miR-34a E1, miR-34a KO, miR-16 E1 and miR-16 KO. E1 represents a GoF and KO represents LoF of the corresponding element. Black box, Ectopic expression (E1) of miR-34a induce bistability in response to DNA Damage and regulates miR-16 and p21 expression. Whereas, Knockout (KO) of miR-34a destroyed the bistability and directly affect miR-16 and p21 expression. On the other hand, Ectopic expression (E1) and/or Knockout (KO) of miR-16 could not disrupt the bistability and/or not affect miR-34a activity. Left-most column lists DNA Damage levels highlighted in orange and right-most columns present the outputs in green: Proliferation, Senescence and Apoptosis. Each line represents a single stable state corresponding to the input. Yellow cells denote a null value. Whereas, red cells denote activation (value 1). (**B**) The prediction about miR-34 and miR-16 in Fig. 2A is consistent with experimental data^2,5,22,39^.

Interestingly, we found that miR-34a can regulate miR-16 expression, see Fig. 2A. It is evident that inhibition of miR-16 by HDAC1^2^ or c-Myc^39,40^ increases its expression and its well known that HDAC1 and/or c-Myc is a direct target of miR-34a. Thus, miR-34a can regulate miR-16 expression. We found that knockdown of miR-34a diminishes miR-16 levels. Whereas, overexpression of miR-34a increases miR-16 levels. In this way, miR-34a can regulate miR-16 expression in NSCLC cells and possibly in CTCL cells, too (see Fig. 2B). Furthermore, we also found that RB can regulate miR-16 expression in agreement with the study of Bandi *et al*.^4^, see Fig. 2.

### Computational-experimental integration of the model for NSCLC and CTCL cells

**Figure 3:**
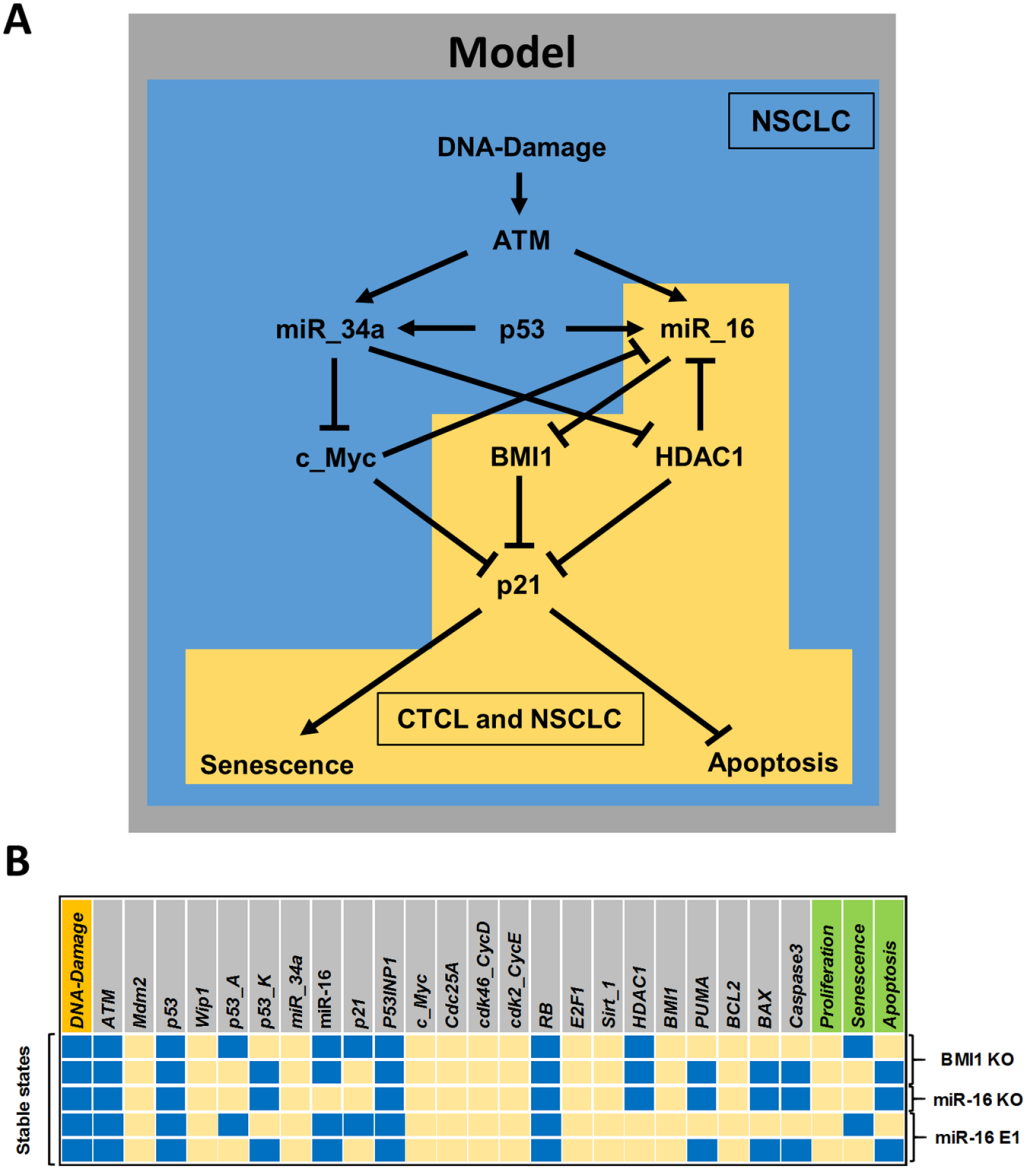
Correlation of molecular mechanisms in CTCL and NSCLC and corresponding simulation results. (**A**) The model network (background in gray) contains the miR-16 downstream network in CTCL (yellow background) that composes the cell fate decision pathway in NSCLC (mixed blue and yellow background). (**B**) Model validation of the stable states of the model according to experimental perturbations for CTCLs^2^.

In order to integrate the model for NSCLC with other cellular types, we performed an extensive bibliographical research about the influence in cell fate of other cell lines sensitive to miR-34 and/or miR-16. In this way, we found that the study proposed by Kitadate and colleagues^2^ demonstrated that the treatment with HDAC inhibitors (SAHA) in CTCL cells significantly increased miR-16 expression. They further showed that p21 is inhibited by BMI1, which is also a target of miR-16. In this way, they showed that SAHA increases miR-16 expression, indirectly upregulating p21 expression via targeting of BMI1. Thus, p21 acts as a switch-like behavior controlling the cell fate decision between senescence and apoptosis in CTCL cells. The downstream effect of miR-16 observed in the study proposed by Kitadate and colleagues^2^ is exactly the same observed in studies for NSCLC cells (see Fig. 3A). Thus, it allows us to compare the model results with experimental observations by Kitadate and colleagues^2^. Our model reproduces all experimental results of such study (Fig. 3B). In this way, we suggest that miR-16-responsive cell fate decision in NSCLC and CTCL cells may have the same control. However, the LoF of miR-34a (see Fig. 2) in our model could not reproduce similar results, suggesting that the miR-34a influence on cell fate decision in CTCL cells is different.

### Suggested experimental designs

The idea of experimental designs is motivated by the work by Cyrenne *et al*.^41^ and Kim *et al*.^42^ that show that the combined knockdown of BCL2 and HDAC1^41^ can be a promising treatment of CTCL. These studies led us to propose a new experimental design based on our model, which consists in using miR-34a as an inhibitor of HDAC1/BCL2/c-Myc pathways. The possible outcome of this experiment would be a functionally stable p21 and/or miR-16. To test this outcome, we propose these possible strategies predicted by the model:

- miR-34a [E1], HDAC1 [KO] → p21
- miR-34a [E1], c-Myc [KO] → p21
- miR-34a [E1], HDAC1 [KO] → miR-16
- miR-34a [E1], c-Myc [KO] → miR-16

## Discussion and Conclusions

MicroRna’s 34a and 16 are regulators of a variety of different molecules involved in cell cycle progression mechanisms, such as CDK4 and CDK6. In this context, Bandi and Vassella^1^ demonstrated that both microRNAs can control the G1/S cell cycle checkpoint induction in a synergistic manner in NSCLC cells potentiating their impact on the G1/S progression. In addition to these evidences, studies showed that both miRNAs can also regulate phenotypes in NSCLC cells in an individual manner^4,5^. Bandi *et al*.^4^ observed that the overexpression of miR-16 can induce cell cycle arrest in NSCLC cells. In miR-34a context, several studies demonstrated that this molecule can regulate the fate decision between senescence and apoptosis in NSCLC cells. However, the extension to other cell types of the role of miR-16 and miR-34a is still unclear. A recent study showed that miR-16 can regulate the senescence-apoptosis switch in CTCL cells;^2^ however no other study in the literature investigated the role of miR-34 on these cells. Thus, in the present study we integrated in a computational model the influence on cell fate of miR-16 and miR-34a for NSCLC cells based on the work by Bandi and Vassella’s^1^. In this way, we observed that miR-16 in NSCLC cells presents similar role in the G1/S checkpoint induction as in CTCL cells. This finding allows us to predict that the mechanisms recognized for miR-34a in NSCLC cells might also be observed in CTCL cells.

Recent *in vivo* studies have demonstrated that miR-16 and miR-34a can act as antiproliferative agents in NSCLC cell lines, suggesting their potential as therapeutic targets for NSCLC^43–46^. Their high impact on the regulation of cell cycle progression is strongly related to a variety of different molecules that both regulate. Moreover, the synergistic effect between miR-34a and miR-16 can amplify their global role in cell cycle regulation. Bandi and Vassella^1^ demonstrated that such miRNAs can act synergistically to regulate cell cycle arrest in NSCLC. In addition, a recent study proposed by Orellana and colleagues^43^ has shown that miR-34a can synergize with other miRNAs to act as antiproliferative agents in NSCLC cell lines, demonstrating that the link between miR-16 and miR-34a is not unique and can occur with other miRNAs in NSCLC. Our study focused on miR-16 and miR-34a influence according to available experimental studies and highlight some unrecognized mechanisms involving these two miRNAs. However, it is not excluded the possibility that other miRNAs might present an important role in the regulation of cell cycle progression in NSCLC.

To improve the knowledge about the synergistic regulation of cell fate decision in NSCLC cells through miR-34a and miR-16, a mechanistic understanding of this process is indispensable. For that, a computational model was built based on experimental results about the synergistic role of such molecules in the G1/S checkpoint in NSCLC cells. In the absence of DNA damage, the model predicts only a stable state characterized by a proliferative phenotype which is supported by Bandi and Vassella^1^. In the presence of DNA damage, a bistable dynamics was observed, which corresponds to two p53-responsive cellular phenotypes: senescence or apoptosis. Indeed, both phenotypes are observed in NSCLC in DNA damage response^5^. The NSCLC cell model was validated through GoF and LoF perturbations of its components according to experimental results from Bandi and Vassella work^1^ that analyzed the synergistic role of miR-16 and miR-34a in NSCLC cells. The agreement with experimental results of the synergistic model for miR-34a and miR-16 for NSCLC cells reproduces the known experimental results for each microRNA individually.

Our modelling approach allows several experimentally testable predictions. In terms of the perturbations presented in Table 2: E2F1 and Sirt-1 can induce a bistable phenotype between senescence and apoptosis through their downregulation. Indeed, both phenotypes were observed in cells lacking E2F1 or Sirt-1^47,48^. Moreover, our model predicts an important finding in NSCLC and CTCL cells that miR-34a can regulate miR-16 via HDAC1 and/or c-Myc. For that goal we suggested an experimental design where miR-34a targets HDAC1/c-Myc to regulate miR-16 and/or p21 expression.

In summary, our model agrees with experimental results associated with synergistic or individual influence of miR-16 and miR-34a in cell fate decision in NSCLC cells. Such analysis allowed the integration of the dynamical behavior of NSCLC with CTCLs cells via investigation of their corresponding mechanisms regarding miR-34 and miR-16 influence on cell fate decision.

## Supplementary information

Supplementary Information.

Supplementary Information 2.
