# Supplementary material for: Integrative data modeling from lung and lymphatic cancer predicts functional roles for miR-34a and miR-16 in cell fate regulation
Source: Sci Rep. 2020 Feb 13;10:2511. doi: 10.1038/s41598-020-59339-y (PMC7018995; doi:10.1038/s41598-020-59339-y)
Supplement: Supplementary file 2 — Supplementary Information 2. [file 41598_2020_59339_MOESM2_ESM.pdf]

Integrative data modeling from lung and lymphatic cancer predicts functional roles for miR-34a and miR-16 in cell fate regulation
Supplementary file S1

Shantanu Gupta[1,*] | Daner A. Silveira[1,*] | Florencia M. Barbé-Tuana[2,*] and José C. M. Mombach[1,*,+]

[1]Departmento de Física, Universidade Federal de Santa Maria, Santa Maria, RS, 97105-900, Brazil
[2]Postgraduate Program in Cellular and Molecular Biology, School of Sciences, Pontifícia Universidade Católica do Rio Grande do Sul

[+]Corresponding author (e-mail: jcmombach@ufsm.br)
[*]These authors contributed equally to this work

Code of the model in .ginml file format used for the GINsim 3.0.0b software.

```
<?xml version="1.0" encoding="UTF-8"?>
<!DOCTYPE gxl SYSTEM "http://ginsim.org/GINML_2_2.dtd">
<gxl xmlns:xlink="http://www.w3.org/1999/xlink">
  <graph class="regulatory" id="NEW-SYNERGY-MODEL_FORMAT-JUNE-2019" nodeorder="DNA_Damage ATM Mdm2 p53 Wip1 p53_A p53_K miR_34a miR_16 p21 p53INP1 c_Myc Cdc25A CDK46_CycD CDK2_CycE RB E2F1 Sirt_1 HDAC1 BMI1 PUMA BCL2 BAX Caspase3 Poliferation Senescence Apoptosis">
    <nodestyle background="#ffffff" foreground="#000000" text="#000000" shape="RECTANGLE" width="45" height="25" properties="intermediate:#ffff00 active:#ffc800"/>
    <nodestyle name="Node style" background="#ff0000" foreground="#333333" width="90" height="40"/>
    <nodestyle name="Node style 2" background="#ffffff" foreground="#000000" shape="RECTANGLE" width="60" height="30"/>
    <nodestyle name="Node style 3" background="#ffff00" foreground="#000000" text="#000000" width="70" height="30"/>
    <nodestyle name="Node style 4" background="#ffff00" foreground="#000000" text="#000000" width="70" height="30"/>
    <nodestyle name="Node style 5" background="#ffffff" shape="RECTANGLE" width="80" height="30"/>
    <nodestyle name="Node style 6" shape="RECTANGLE" width="80" height="30"/>
    <nodestyle name="Node style 7" background="#33ff33" shape="RECTANGLE" width="80" height="30"/>
    <edgestyle color="#000000" pattern="SIMPLE" line_width="2" properties="positive:#00c800 negative:#c80000 dual:#0000c8"/>
    <edgestyle name="Edge style" pattern="DASH"/>
    <edgestyle name="Edge style 2" pattern="DASH"/>
    <node id="DNA_Damage" maxvalue="1" input="true">
      <nodevisualsetting x="139" y="109" style="Node style"/>
    </node>
    <node id="ATM" maxvalue="1">
      <value val="1">
        <exp str="DNA_Damage &amp; (!HDAC1 | !Wip1 | E2F1)"/>
      </value>
      <nodevisualsetting x="310" y="52" style="Node style 2"/>
    </node>
    <node id="Mdm2" maxvalue="1">
      <value val="1">
        <exp str="(!Wip1 | p53) &amp; !ATM"/>
      </value>
      <nodevisualsetting x="530" y="273" style="Node style 2"/>
    </node>
    <node id="p53" maxvalue="1">
      <value val="1">
        <exp str="(ATM | !Sirt_1) &amp; !Mdm2"/>
      </value>
      <nodevisualsetting x="317" y="274" style="Node style 2"/>
    </node>
    <node id="Wip1" maxvalue="1">
      <value val="1">
        <exp str="p53_A &amp; !miR_16"/>
      </value>
      <nodevisualsetting x="879" y="42" style="Node style 2"/>
    </node>
    <node id="p53_A" maxvalue="1">
      <value val="1">
        <exp str="!Sirt_1 &amp; !p53_K &amp; (p53:1 | !p53INP1)"/>
      </value>
      <nodevisualsetting x="319" y="366" style="Node style 2"/>
    </node>
```

```
<node id="p53_K" maxvalue="1">
  <value val="1">
    <exp str="!p53_A &amp; (!Sirt_1 | !Wip1) &amp; p53"/>
  </value>
  <nodevisualsetting x="528" y="366" style="Node style 2"/>
</node>
<node id="miR_34a" maxvalue="1">
  <value val="1">
    <exp str="ATM | p53"/>
  </value>
  <nodevisualsetting x="328" y="128" style="Node style 3"/>
</node>
<node id="miR_16" maxvalue="1">
  <value val="1">
    <exp str="(ATM | p53) &amp; !HDAC1 &amp; !c_Myc"/>
  </value>
  <nodevisualsetting x="699" y="120" style="Node style 4"/>
</node>
<node id="p21" maxvalue="1">
  <value val="1">
    <exp str="p53_A | ((!HDAC1 | !c_Myc | !BMI1) &amp; !Caspase3)"/>
  </value>
  <nodevisualsetting x="710" y="265" style="Node style 2"/>
</node>
<node id="p53INP1" maxvalue="1">
  <value val="1">
    <exp str="p53_K | p53_A"/>
  </value>
  <nodevisualsetting x="368" y="424" style="Node style 2"/>
</node>
<node id="c_Myc" maxvalue="1">
  <value val="1">
    <exp str="(E2F1 | !miR_34a) &amp; !RB &amp; !p21"/>
  </value>
  <nodevisualsetting x="529" y="170" style="Node style 2"/>
</node>
<node id="Cdc25A" maxvalue="1">
  <value val="1">
    <exp str="!ATM &amp; !miR_34a"/>
  </value>
  <nodevisualsetting x="529" y="466" style="Node style 2"/>
</node>
<node id="CDK46_CycD" maxvalue="1">
  <value val="1">
    <exp str="Cdc25A &amp; !miR_34a &amp; !p21 &amp; !miR_16"/>
  </value>
  <nodevisualsetting x="397" y="541" style="Node style 5"/>
</node>
<node id="CDK2_CycE" maxvalue="1">
  <value val="1">
    <exp str="Cdc25A &amp; E2F1 &amp; !miR_34a &amp; !p21 &amp; !miR_16"/>
  </value>
  <nodevisualsetting x="607" y="541" style="Node style 5"/>
</node>
<node id="RB" maxvalue="1">
  <value val="1">
    <exp str="!CDK46_CycD &amp; !CDK2_CycE"/>
  </value>
  <nodevisualsetting x="323" y="466" style="Node style 2"/>
</node>
<node id="E2F1" maxvalue="1">
  <value val="1">
```

```
        <exp str="(!RB &amp; ((Cdc25A &amp; ATM) | !Sirt_1 | !miR_34a)) | c_Myc"/>
      </value>
      <nodevisualsetting x="721" y="463" style="Node style 2"/>
    </node>
    <node id="Sirt_1" maxvalue="1">
      <value val="1">
        <exp str="E2F1 &amp; !(miR_34a &amp; HDAC1)"/>
      </value>
      <nodevisualsetting x="883" y="460" style="Node style 2"/>
    </node>
    <node id="HDAC1" maxvalue="1">
      <value val="1">
        <exp str="(!miR_34a &amp; !Sirt_1) | !DNA_Damage"/>
      </value>
      <nodevisualsetting x="529" y="46" style="Node style 2"/>
    </node>
    <node id="BMI1" maxvalue="1">
      <value val="1">
        <exp str="!miR_16 &amp; (E2F1 | c_Myc)"/>
      </value>
      <nodevisualsetting x="880" y="163" style="Node style 2"/>
    </node>
    <node id="PUMA" maxvalue="1">
      <value val="1">
        <exp str="p53_K"/>
      </value>
      <nodevisualsetting x="710" y="364" style="Node style 2"/>
    </node>
    <node id="BCL2" maxvalue="1">
      <value val="1">
        <exp str="!PUMA &amp; !miR_34a &amp; !miR_16"/>
      </value>
      <nodevisualsetting x="879" y="263" style="Node style 2"/>
    </node>
    <node id="BAX" maxvalue="1">
      <value val="1">
        <exp str="!BCL2 &amp; (p53_K | c_Myc)"/>
      </value>
      <nodevisualsetting x="880" y="349" style="Node style 2"/>
    </node>
    <node id="Caspase3" maxvalue="1">
      <value val="1">
        <exp str="!(BCL2 &amp; p21) &amp; BAX"/>
      </value>
      <nodevisualsetting x="869" y="537" style="Node style 5"/>
    </node>
    <node id="Poliferation" maxvalue="1">
      <value val="1">
        <exp str="CDK2_CycE"/>
      </value>
      <nodevisualsetting x="998" y="335" style="Node style 7"/>
    </node>
    <node id="Senescence" maxvalue="1">
      <value val="1">
        <exp str="p21 &amp; !CDK2_CycE"/>
      </value>
      <nodevisualsetting x="1002" y="427" style="Node style 7"/>
    </node>
    <node id="Apoptosis" maxvalue="1">
      <value val="1">
        <exp str="Caspase3"/>
      </value>
```

```xml
      <nodevisualsetting x="1004" y="535" style="Node style 7"/>
    </node>
    <edge id="c_Myc:miR_16" from="c_Myc" to="miR_16" minvalue="1" sign="negative">
      <edgevisualsetting points="558,148" anchor="NE" style=""/>
    </edge>
    <edge id="c_Myc:p21" from="c_Myc" to="p21" minvalue="1" sign="negative">
      <edgevisualsetting points="568,265" anchor="NE" style=""/>
    </edge>
    <edge id="c_Myc:BAX" from="c_Myc" to="BAX" minvalue="1" sign="positive">
      <edgevisualsetting points="858,180 858,346" anchor="NE" style=""/>
    </edge>
    <edge id="c_Myc:E2F1" from="c_Myc" to="E2F1" minvalue="1" sign="positive">
      <edgevisualsetting points="474,174 474,444 745,444" anchor="NE" style=""/>
    </edge>
    <edge id="c_Myc:BMI1" from="c_Myc" to="BMI1" minvalue="1" sign="positive">
      <edgevisualsetting points="574,164" anchor="NE" style=""/>
    </edge>
    <edge id="p53:miR_34a" from="p53" to="miR_34a" minvalue="1" sign="positive">
      <edgevisualsetting points="344,230" anchor="NE" style=""/>
    </edge>
    <edge id="p53:miR_16" from="p53" to="miR_16" minvalue="1" sign="positive">
      <edgevisualsetting points="356,244 731,244" anchor="NE" style=""/>
    </edge>
    <edge id="p53:Mdm2" from="p53" to="Mdm2" minvalue="1" sign="positive">
      <edgevisualsetting points="418,295" anchor="NE" style=""/>
    </edge>
    <edge id="p53:p53_A" from="p53" to="p53_A" minvalue="1" sign="positive">
      <edgevisualsetting points="327,339" anchor="NE" style=""/>
    </edge>
    <edge id="p53:p53_K" from="p53" to="p53_K" minvalue="1" sign="positive">
      <edgevisualsetting points="351,331 562,331" anchor="NE" style=""/>
    </edge>
    <edge id="BMI1:p21" from="BMI1" to="p21" minvalue="1" sign="negative">
      <edgevisualsetting points="764,174" anchor="NE" style=""/>
    </edge>
    <edge id="CDK46_CycD:RB" from="CDK46_CycD" to="RB" minvalue="1" sign="negative">
      <edgevisualsetting points="347,541" anchor="NE" style=""/>
    </edge>
    <edge id="Caspase3:p21" from="Caspase3" to="p21" minvalue="1" sign="negative">
      <edgevisualsetting points="811,546 811,318 763,318" anchor="NE" style=""/>
    </edge>
    <edge id="Caspase3:Apoptosis" from="Caspase3" to="Apoptosis" minvalue="1" sign="positive">
      <edgevisualsetting points="974,554" anchor="NE" style=""/>
    </edge>
    <edge id="Wip1:ATM" from="Wip1" to="ATM" minvalue="1" sign="negative">
      <edgevisualsetting points="910,32 361,32" anchor="NE" style=""/>
    </edge>
    <edge id="Wip1:Mdm2" from="Wip1" to="Mdm2" minvalue="1" sign="negative">
      <edgevisualsetting points="888,110 819,110 819,204 610,204 610,291" anchor="NE" style=""
          />
    </edge>
    <edge id="Wip1:p53_K" from="Wip1" to="p53_K" minvalue="1" sign="negative">
      <edgevisualsetting points="799,68 799,329 603,329 603,374" anchor="NE" style=""/>
    </edge>
    <edge id="HDAC1:miR_16" from="HDAC1" to="miR_16" minvalue="1" sign="negative">
      <edgevisualsetting points="557,141" anchor="NE" style=""/>
    </edge>
    <edge id="HDAC1:ATM" from="HDAC1" to="ATM" minvalue="1" sign="negative">
      <edgevisualsetting points="425,63" anchor="NE" style=""/>
    </edge>
    <edge id="HDAC1:Sirt_1" from="HDAC1" to="Sirt_1" minvalue="1" sign="negative">
      <edgevisualsetting points="499,74 499,409 922,409" anchor="NE" style=""/>
```

```
    </edge>
    <edge id="HDAC1:p21" from="HDAC1" to="p21" minvalue="1" sign="negative">
      <edgevisualsetting points="790,69 790,276" anchor="NE" style=""/>
    </edge>
    <edge id="RB:c_Myc" from="RB" to="c_Myc" minvalue="1" sign="negative">
      <edgevisualsetting points="436,481 436,187" anchor="NE" style=""/>
    </edge>
    <edge id="RB:E2F1" from="RB" to="E2F1" minvalue="1" sign="negative">
      <edgevisualsetting points="363,512 756,512" anchor="NE" style=""/>
    </edge>
    <edge id="DNA_Damage:ATM" from="DNA_Damage" to="ATM" minvalue="1" sign="positive">
      <edgevisualsetting points="202,61" anchor="NE" style=""/>
    </edge>
    <edge id="DNA_Damage:HDAC1" from="DNA_Damage" to="HDAC1" minvalue="1" sign="negative">
      <edgevisualsetting points="184,12 555,12" anchor="NE" style=""/>
    </edge>
    <edge id="CDK2_CycE:RB" from="CDK2_CycE" to="RB" minvalue="1" sign="negative">
      <edgevisualsetting points="627,580 335,580" anchor="NE" style=""/>
    </edge>
    <edge id="CDK2_CycE:Poliferation" from="CDK2_CycE" to="Poliferation" minvalue="1" sign="positive">
      <edgevisualsetting points="755,559 754,587 967,586 966,354" anchor="NE" style=""/>
    </edge>
    <edge id="CDK2_CycE:Senescence" from="CDK2_CycE" to="Senescence" minvalue="1" sign="negative">
      <edgevisualsetting points="793,555 793,515 977,516 977,453" anchor="NE" style=""/>
    </edge>
    <edge id="E2F1:ATM" from="E2F1" to="ATM" minvalue="1" sign="positive">
      <edgevisualsetting points="737,587 307,587" anchor="NE" style=""/>
    </edge>
    <edge id="E2F1:c_Myc" from="E2F1" to="c_Myc" minvalue="1" sign="positive">
      <edgevisualsetting points="654,478 654,185" anchor="NE" style=""/>
    </edge>
    <edge id="E2F1:Sirt_1" from="E2F1" to="Sirt_1" minvalue="1" sign="positive">
      <edgevisualsetting points="831,474" anchor="NE" style=""/>
    </edge>
    <edge id="E2F1:CDK2_CycE" from="E2F1" to="CDK2_CycE" minvalue="1" sign="positive">
      <edgevisualsetting points="730,548" anchor="NE" style=""/>
    </edge>
    <edge id="E2F1:BMI1" from="E2F1" to="BMI1" minvalue="1" sign="positive">
      <edgevisualsetting points="823,491 823,223 924,223" anchor="NE" style=""/>
    </edge>
    <edge id="BCL2:BAX" from="BCL2" to="BAX" minvalue="1" sign="negative">
      <edgevisualsetting points="906,326" anchor="NE" style=""/>
    </edge>
    <edge id="BCL2:Caspase3" from="BCL2" to="Caspase3" minvalue="1" sign="negative">
      <edgevisualsetting points="834,290 834,525 881,525" anchor="NE" style=""/>
    </edge>
    <edge id="BAX:Caspase3" from="BAX" to="Caspase3" minvalue="1" sign="positive">
      <edgevisualsetting points="843,371 843,539" anchor="NE" style=""/>
    </edge>
    <edge id="ATM:miR_34a" from="ATM" to="miR_34a" minvalue="1" sign="positive">
      <edgevisualsetting points="355,105" anchor="NE" style=""/>
    </edge>
    <edge id="ATM:miR_16" from="ATM" to="miR_16" minvalue="1" sign="positive">
      <edgevisualsetting points="349,21 731,21" anchor="NE" style=""/>
    </edge>
    <edge id="ATM:p53" from="ATM" to="p53" minvalue="1" sign="positive">
      <edgevisualsetting points="320,117" anchor="NE" style=""/>
    </edge>
    <edge id="ATM:Mdm2" from="ATM" to="Mdm2" minvalue="1" sign="negative">
      <edgevisualsetting points="487,75 487,275" anchor="NE" style=""/>
```

```
</edge>
<edge id="ATM:Cdc25A" from="ATM" to="Cdc25A" minvalue="1" sign="negative">
  <edgevisualsetting points="466,81 466,467" anchor="NE" style=""/>
</edge>
<edge id="ATM:E2F1" from="ATM" to="E2F1" minvalue="1" sign="positive">
  <edgevisualsetting points="248,72 248,523 762,523" anchor="NE" style=""/>
</edge>
<edge id="miR_16:Wip1" from="miR_16" to="Wip1" minvalue="1" sign="negative">
  <edgevisualsetting points="909,130" anchor="NE" style=""/>
</edge>
<edge id="miR_16:BMI1" from="miR_16" to="BMI1" minvalue="1" sign="negative">
  <edgevisualsetting points="911,137" anchor="NE" style=""/>
</edge>
<edge id="miR_16:BCL2" from="miR_16" to="BCL2" minvalue="1" sign="negative">
  <edgevisualsetting points="745,211 915,211" anchor="NE" style=""/>
</edge>
<edge id="miR_16:CDK46_CycD" from="miR_16" to="CDK46_CycD" minvalue="1" sign="negative">
  <edgevisualsetting points="710,211 444,211" anchor="NE" style=""/>
</edge>
<edge id="miR_16:CDK2_CycE" from="miR_16" to="CDK2_CycE" minvalue="1" sign="negative">
  <edgevisualsetting points="719,215 682,215" anchor="NE" style=""/>
</edge>
<edge id="PUMA:BCL2" from="PUMA" to="BCL2" minvalue="1" sign="negative">
  <edgevisualsetting points="829,377 829,278" anchor="NE" style=""/>
</edge>
<edge id="p53_A:Wip1" from="p53_A" to="Wip1" minvalue="1" sign="positive">
  <edgevisualsetting points="342,349 843,349 843,49" anchor="NE" style=""/>
</edge>
<edge id="p53_A:p53_K" from="p53_A" to="p53_K" minvalue="1" sign="negative">
  <edgevisualsetting anchor="NE" style=""/>
</edge>
<edge id="p53_A:p21" from="p53_A" to="p21" minvalue="1" sign="positive">
  <edgevisualsetting points="334,340 729,340" anchor="NE" style=""/>
</edge>
<edge id="p53_A:p53INP1" from="p53_A" to="p53INP1" minvalue="1" sign="positive">
  <edgevisualsetting points="407,382" anchor="NE" style=""/>
</edge>
<edge id="p53_K:p53_A" from="p53_K" to="p53_A" minvalue="1" sign="negative">
  <edgevisualsetting points="420,391" anchor="NE" style=""/>
</edge>
<edge id="p53_K:PUMA" from="p53_K" to="PUMA" minvalue="1" sign="positive">
  <edgevisualsetting points="628,380" anchor="NE" style=""/>
</edge>
<edge id="p53_K:BAX" from="p53_K" to="BAX" minvalue="1" sign="positive">
  <edgevisualsetting points="575,356" anchor="NE" style=""/>
</edge>
<edge id="p53_K:p53INP1" from="p53_K" to="p53INP1" minvalue="1" sign="positive">
  <edgevisualsetting points="542,424 487,424 487,473 404,473" anchor="NE" style=""/>
</edge>
<edge id="Sirt_1:HDAC1" from="Sirt_1" to="HDAC1" minvalue="1" sign="negative">
  <edgevisualsetting points="899,455 851,455 849,61" anchor="NE" style=""/>
</edge>
<edge id="Sirt_1:p53" from="Sirt_1" to="p53" minvalue="1" sign="negative">
  <edgevisualsetting points="907,450 641,450 641,345 507,345 507,307" anchor="NE" style=""
      />
</edge>
<edge id="Sirt_1:E2F1" from="Sirt_1" to="E2F1" minvalue="1" sign="negative">
  <edgevisualsetting points="832,483" anchor="NE" style=""/>
</edge>
<edge id="Sirt_1:p53_A" from="Sirt_1" to="p53_A" minvalue="1" sign="negative">
  <edgevisualsetting points="912,415 338,415" anchor="NE" style=""/>
</edge>
```

```xml
    <edge id="Sirt_1:p53_K" from="Sirt_1" to="p53_K" minvalue="1" sign="negative">
      <edgevisualsetting points="908,423 605,423 605,387" anchor="NE" style=""/>
    </edge>
    <edge id="p53INP1:p53_A" from="p53INP1" to="p53_A" minvalue="1" sign="negative">
      <edgevisualsetting points="353,436" anchor="NE" style=""/>
    </edge>
    <edge id="Cdc25A:CDK46_CycD" from="Cdc25A" to="CDK46_CycD" minvalue="1" sign="positive">
      <edgevisualsetting points="467,478" anchor="NE" style=""/>
    </edge>
    <edge id="Cdc25A:CDK2_CycE" from="Cdc25A" to="CDK2_CycE" minvalue="1" sign="positive">
      <edgevisualsetting points="638,478" anchor="NE" style=""/>
    </edge>
    <edge id="Cdc25A:E2F1" from="Cdc25A" to="E2F1" minvalue="1" sign="positive">
      <edgevisualsetting points="676,485" anchor="NE" style=""/>
    </edge>
    <edge id="p21:c_Myc" from="p21" to="c_Myc" minvalue="1" sign="negative">
      <edgevisualsetting points="739,194" anchor="NE" style=""/>
    </edge>
    <edge id="p21:CDK46_CycD" from="p21" to="CDK46_CycD" minvalue="1" sign="negative">
      <edgevisualsetting points="670,280 670,463 623,463 623,517 570,517 570,555" anchor="NE"
          style=""/>
    </edge>
    <edge id="p21:CDK2_CycE" from="p21" to="CDK2_CycE" minvalue="1" sign="negative">
      <edgevisualsetting points="676,286" anchor="NE" style=""/>
    </edge>
    <edge id="p21:Caspase3" from="p21" to="Caspase3" minvalue="1" sign="negative">
      <edgevisualsetting points="807,285 807,554" anchor="NE" style=""/>
    </edge>
    <edge id="p21:Senescence" from="p21" to="Senescence" minvalue="1" sign="positive">
      <edgevisualsetting points="747,324 973,325 974,442" anchor="NE" style=""/>
    </edge>
    <edge id="miR_34a:HDAC1" from="miR_34a" to="HDAC1" minvalue="1" sign="negative">
      <edgevisualsetting points="549,142" anchor="NE" style=""/>
    </edge>
    <edge id="miR_34a:c_Myc" from="miR_34a" to="c_Myc" minvalue="1" sign="negative">
      <edgevisualsetting points="550,149" anchor="NE" style=""/>
    </edge>
    <edge id="miR_34a:E2F1" from="miR_34a" to="E2F1" minvalue="1" sign="negative">
      <edgevisualsetting points="373,238 818,238 817,467" anchor="NE" style=""/>
    </edge>
    <edge id="miR_34a:BCL2" from="miR_34a" to="BCL2" minvalue="1" sign="negative">
      <edgevisualsetting points="362,229 901,229" anchor="NE" style=""/>
    </edge>
    <edge id="miR_34a:Cdc25A" from="miR_34a" to="Cdc25A" minvalue="1" sign="negative">
      <edgevisualsetting points="269,159 268,517 537,517" anchor="NE" style=""/>
    </edge>
    <edge id="miR_34a:CDK46_CycD" from="miR_34a" to="CDK46_CycD" minvalue="1" sign="negative">
      <edgevisualsetting points="288,152 287,551" anchor="NE" style=""/>
    </edge>
    <edge id="miR_34a:CDK2_CycE" from="miR_34a" to="CDK2_CycE" minvalue="1" sign="negative">
      <edgevisualsetting points="383,253 663,253" anchor="NE" style=""/>
    </edge>
    <edge id="miR_34a:Sirt_1" from="miR_34a" to="Sirt_1" minvalue="1" sign="negative">
      <edgevisualsetting points="398,220 837,220 837,467" anchor="NE" style=""/>
    </edge>
    <edge id="Mdm2:p53" from="Mdm2" to="p53" minvalue="1" sign="negative">
      <edgevisualsetting points="484,283" anchor="NE" style=""/>
    </edge>
  </graph>
</gxl>
```
